# Supplementary material for: Spatial transcriptomics in bone mechanomics: Exploring the mechanoregulation of fracture healing in the era of spatial omics
Source: Sci Adv. 2025 Jan 1;11(1):eadp8496. doi: 10.1126/sciadv.adp8496 (PMC11694773; doi:10.1126/sciadv.adp8496)
Supplement: Supplementary file 1 — Figs. S1 and S2 Legends for tables S1 to S6 [file sciadv.adp8496_sm.pdf]

Science Advances
AAAS

Supplementary Materials for

Spatial transcriptomics in bone mechanomics: Exploring the mechanoregulation of fracture healing in the era of spatial omics

Neashan Mathavan *et al.*

Corresponding author: Ralph Müller, ram@ethz.ch

Sci. Adv. **11**, eadp8496 (2025)
DOI: 10.1126/sciadv.adp8496

The PDF file includes:

Figs. S1 and S2
Legends for tables S1 to S6

Other Supplementary Material for this manuscript includes the following:

Tables S1 to S6

Supplementary Materials

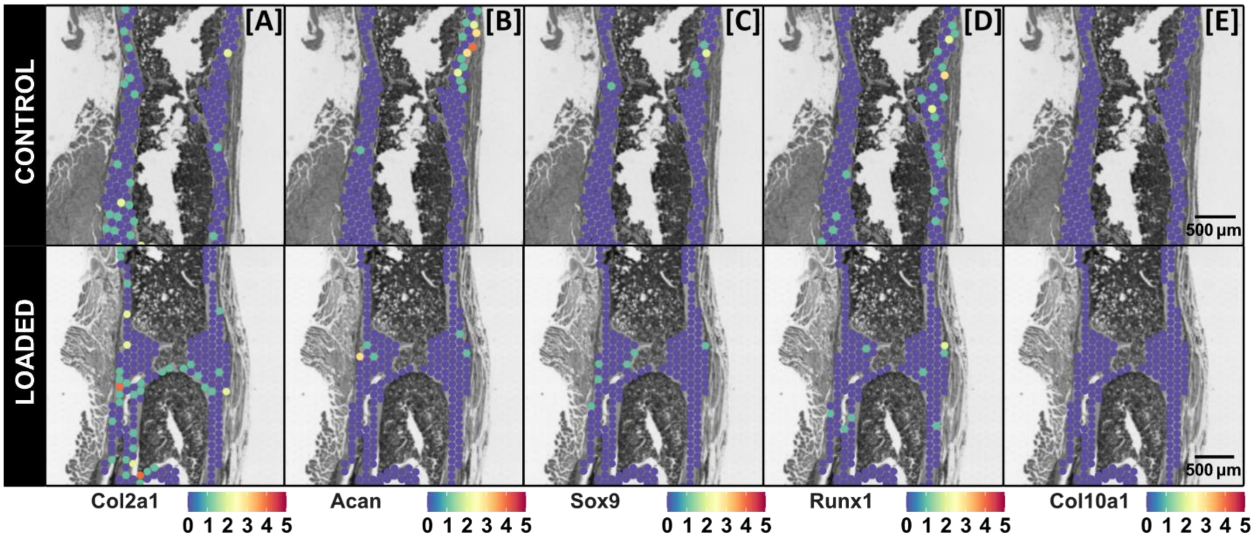

Supplementary Fig. S1. Spatial gene expression maps of selected chondrocyte markers at the fracture site of Control and Loaded mice. Visualization of the spatial expression patterns of chondrocyte markers: **[A]** *Col2a1*, **[B]** *Acan*, **[C]** *Sox9*, **[D]** *Runx1* and **[E]** *Col10a1* within the fracture sites of Control and Loaded mice are presented. Each legend denotes the normalized expression of each gene. Data presented (n = 1 per group) corresponds to samples at 5 weeks post-surgery. 3D visualizations of the morphology of these Control and Loaded fracture sites are presented in Figure 2. Spatial transcriptomics spot size = 55 µm.

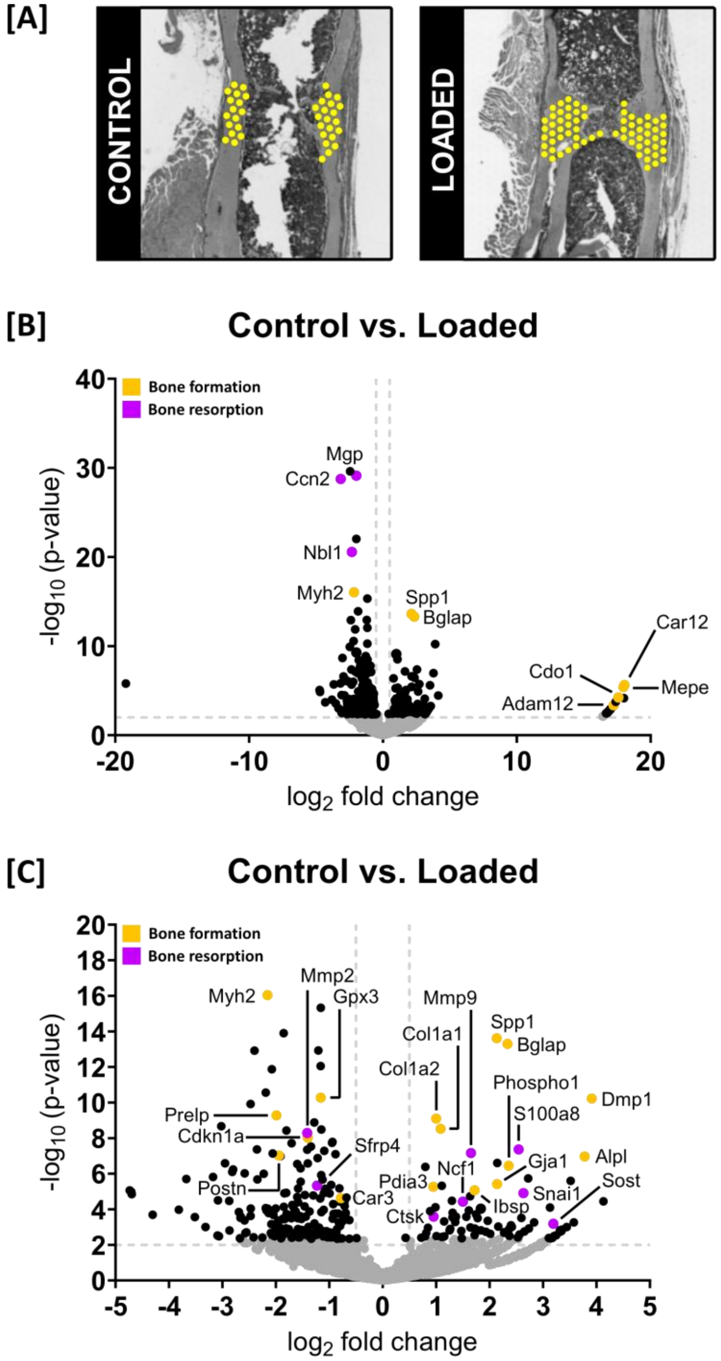

Supplementary Fig. S2. Gene expression profiling in DC + DP regions of Control vs. Loaded fracture sites. Data presented (n = 1 per group) corresponds to samples at 5 weeks post-surgery. **[A]**: Areas of interest defined in each fracture site for the analysis. **[B]**: Volcano plot to visualize differentially expressed genes (DEGs) (significance criteria: FDR-adjusted p value cutoff < 0.05 and an absolute log2 fold change > 0.5). Significant DEGs associated with bone formation are identified in orange and significant DEGs associated

with bone resorption (or are inhibitors / antagonists of bone formation) are identified in purple. Non-DEGs are represented in gray. **[C]**: Magnified view of volcano plot to highlight DEGs of interest.

Supplementary Table S1. Barcodes corresponding to all spots of the Control and Loaded bone fracture sites.

Supplementary Table S2. Differential gene expression analyses (DEG) between Control and Loaded fracture sites.

Supplementary Table S3. Differential gene expression (DEG) analysis of high strain region with respect to reference region.

Supplementary Table S4. Differential gene expression (DEG) analysis of low strain region with respect to reference region.

Supplementary Table S5. Gene-set enrichment analysis between mean expression of high strain versus reference strain regions.

Supplementary Table S6. Gene-set enrichment analysis between mean expression of low strain versus reference strain regions.
